# The Application of HEXS and HERFD XANES for Accurate Structural Characterisation of Actinide Nanomaterials: The Case of ThO_2_

**DOI:** 10.1002/chem.202003360

**Published:** 2020-11-12

**Authors:** Lucia Amidani, Gavin B. M. Vaughan, Tatiana V. Plakhova, Anna Yu. Romanchuk, Evgeny Gerber, Roman Svetogorov, Stephan Weiss, Yves Joly, Stepan N. Kalmykov, Kristina O. Kvashnina

**Affiliations:** ^1^ The Rossendorf Beamline at ESRF The European Synchrotron CS40220 38043 Grenoble Cedex 9 France; ^2^ Institute of Resource Ecology Helmholtz Zentrum Dresden-Rossendorf (HZDR), PO Box 510119 01314 Dresden Germany; ^3^ ESRF—The European Synchrotron CS40220 38043 Grenoble Cedex 9 France; ^4^ Department of Chemistry Lomonosov Moscow State University 119991 Moscow Russia; ^5^ National Research Centre “Kurchatov Institute” 123182 Moscow Russia; ^6^ CNRS, Grenoble INP Institut Néel Université Grenoble Alpes 38042 Grenoble France

**Keywords:** actinides, nanoparticles, thorium, X-ray absorption spectroscopy, X-ray scattering

## Abstract

The structural characterisation of actinide nanoparticles (NPs) is of primary importance and hard to achieve, especially for non‐homogeneous samples with NPs less than 3 nm. By combining high‐energy X‐ray scattering (HEXS) and high‐energy‐resolution fluorescence‐detected X‐ray absorption near‐edge structure (HERFD XANES) analysis, we have characterised for the first time both the short‐ and medium‐range order of ThO_2_ NPs obtained by chemical precipitation. By using this methodology, a novel insight into the structures of NPs at different stages of their formation has been achieved. The pair distribution function revealed a high concentration of ThO_2_ small units similar to thorium hexamer clusters mixed with 1 nm ThO_2_ NPs in the initial steps of formation. Drying the precipitates at around 150 °C promoted the recrystallisation of the smallest units into more thermodynamically stable ThO_2_ NPs. HERFD XANES analysis at the thorium M_4_ edge, a direct probe for f states, showed variations that we have correlated with the breakdown of the local symmetry around the thorium atoms, which most likely concerns surface atoms. Together, HEXS and HERFD XANES are a powerful methodology for investigating actinide NPs and their formation mechanism.

## Graphical abstract

**A valuable insight**. We report here a new insight into the formation mechanism of ThO_2_ nanoparticles, obtained by combining high‐energy X‐ray scattering (HEXS) and high‐energy‐resolution fluorescence‐detected X‐ray absorption near‐edge structure (HERFD XANES) analysis methods (see figure). The methodology used here has great potential in the field of actinide nanoparticles.

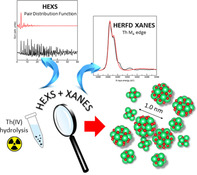

## Introduction

The investigation of nanoscale actinide materials is emerging as a fascinating field of research, challenged by fundamental questions concerning their formation mechanism, interaction with the environment, migration capabilities, fundamental properties and chemical stability.[^1^, ^2^] Despite the fact that nanotechnology has been rapidly developing since the late 20th century and NPs are nowadays ubiquitous in many fields of science, the stage has been dominated by d‐block systems. The f‐block systems, in particular actinides, have been left behind, to the point that to date the properties of nanoscale actinide materials remain largely unknown. The proven tendency of actinides to aggregate in colloidal nanoparticles, which are responsible for their environmental behaviour,^[2]^ calls for an in‐depth understanding of their properties as nanoclusters and nanoparticles, which can present special behaviour, reactivity and structure. Moreover, the high specific surface area of nanosized systems can find application in the design of high burn‐up nuclear fuels.^[3]^ The need for specialised facilities makes actinide research difficult and expensive. On the other hand, the increasing interest in actinides is promoting collaborations among universities, national laboratories, large‐scale research facilities and industries, and important progress has been made. The many gaps and challenges of actinide nanoscience have been recently addressed more systematically owing to the increasing ability to control NP synthesis.[^4^, ^5^, ^6^, ^7^, ^8^, ^9^, ^10^, ^11^] In the roadmap to study NPs, mastering their synthesis goes hand in hand with the ability to accurately characterise the structures of the products. For actinide NPs, a field in its infancy, improvements in the structural characterisation of non‐homogeneous samples would enormously accelerate the understanding of the systems studied.

One of the most investigated topics of radiochemistry at the nanoscale is the formation of tetravalent actinide oxide NPs in aqueous solution.[^7^, ^8^, ^9^, ^12^, ^13^, ^14^, ^15^, ^16^, ^17^] Tracking their aggregation mechanism under different chemical conditions, identifying the presence of multiple oxidation states and characterising their surfaces are real challenges. Even what is considered the simplest system, ThO_2_, for which only the tetravalent oxidation state is stable, is hotly debated. Th^IV^ is the softest among the tetravalent actinide ions and its tendency to hydrolyse is lower than that of other An^IV^. Th^IV^ in solution can form not only mononuclear hydrolysis complexes, but also a number of polynuclear species.[^18^, ^19^, ^20^, ^21^, ^22^, ^23^] The fluorite structure of ThO_2_ is the ultimate product of Th^IV^ hydrolysis, but its well‐defined structure is often identified only after thermal treatment or as a result of ageing processes. Despite attempts to characterise Th^IV^ precipitates since the 1960s,^[24]^ the information on the structure and consequently on ThO_2_ formation mechanisms in solution remains scarce. In most cases, highly hydrolysed thorium salts form Th^IV^ precipitates with ill‐defined structures. In previous studies, such precipitates were classified as amorphous and denoted “Th(OH)_4_(am)” or as hydrous oxides “ThO_2_ **⋅** *x*H_2_O(am)” or “ThO_2_(am,hyd)”, and their amorphous character was identified only by the absence of peaks in their powder XRD (PXRD) patterns.[^25^, ^26^, ^27^] The short‐range local structures of amorphous and crystalline Th^IV^ precipitates were investigated by extended X‐ray absorption fine structure (EXAFS) analysis by Rothe et al.,^[28]^ who first found that in amorphous samples the first Th–O shell is compatible with bond lengths heavily scattered around the values of crystalline ThO_2_. Apart from the evidence of local disorder and the absence of long‐range order, almost no up‐to‐date structural information is available on Th^IV^ hydrous oxide.

Small crystallites of ThO_2_ have seldom been identified in the precipitates obtained under synthesis conditions compatible with the formation of amorphous Th^IV^ hydrous oxide.[^9^, ^29^, ^30^, ^31^, ^32^] Magini et al.^[31]^ investigated hydrolysed thorium salts with wide‐ and small‐angle X‐ray scattering and found small clusters of atoms and microcrystalline ThO_2_ particles up to 4 nm in heat‐treated solutions at relatively mild temperatures (below 100 °C). Dzimitrowicz et al.^[32]^ observed ThO_2_ crystallites of more than 3 nm in X‐ray‐amorphous precipitates by using TEM and electron diffraction. Overall, the nature of Th^IV^ precipitates in aqueous solution remains highly debated because of the absence of a clear‐cut structural characterisation of the products formed, which can be a mixture of different phases difficult to isolate. One way to solve the controversy would be to obtain monodisperse NPs, a goal that up to now has been achieved by using surfactants[^5^, ^33^, ^34^, ^35^] or the pores of a covalent organic framework as an inert template.^[14]^ In the first case, strong binding ligands deriving from organic acids alter the energetics of the surface^[36]^ and ultimately give ThO_2_ NPs of a given morphology and size. In the second case, Moreau et al. obtained monodisperse ThO_2_ NPs below 3 nm and were able to structurally characterise the NPs by X‐ray absorption near‐edge structure (XANES) and EXAFS analysis methods. They found a fluorite structure with substantial local disorder at the surface without the need to invoke an amorphous phase.^[14]^ In all cases, the synthesis routes used different organic thorium precursors, so verification of the ThO_2_ sample produced by chemical precipitation in aqueous media was required. Ultimately, the debate around ThO_2_ and more generally the study of actinide NP formation highlight the need for structural characterisation tools able to probe both short‐ and medium‐range order in solids and liquids.[^37^, ^38^] Indeed, it would be ideal to measure samples without altering their state after synthesis and to characterise all relevant length scales of the system. Up to now, EXAFS has been the structural technique of preference to determine anomalies in the local coordination of actinide NPs compared with the bulk. However, it only provides information on the closest coordination shells.

High‐energy X‐ray scattering (HEXS) and XANES in the hard X‐ray regime comply with these requirements and present specific advantages when applied to actinides. HEXS is among the most powerful techniques applied for the structural investigation of nanomaterials.^[39]^ It measures the arrangement of atoms with Ångström resolution without requiring long‐range order, thereby making it suitable for the characterisation of amorphous and nanostructured systems.[^40^, ^41^] HEXS typically uses the pair distribution function (PDF), which is an appropriately normalised Fourier transform of the scattering signal and provides the probability of finding a pair of atoms separated by a distance *r*. When applied to actinide materials, HEXS provides actinide‐centric pair correlations due to the huge scattering power difference between the metal and the anion, as well as an optimal contrast with the solvent. Soderholm and co‐workers were the first to make systematic use of HEXS to investigate the structures of actinide hydrolysis and condensation products, and to promote its use in actinide research.[^23^, ^37^, ^42^, ^43^, ^44^] Despite their notable results, the application of HEXS to actinide systems remains limited and focused on subnano systems having only a few coordination shells. To the best of our knowledge, we provide here the first in‐depth analysis of HEXS data on heterogeneous samples containing actinide NPs on the nano‐ and subnanoscale.

XANES is also a very powerful tool for the study of nanomaterials.[^45^, ^46^] Its high sensitivity to the local electronic structure of a selected species is very appealing for the study of surface atoms: the sudden break of periodicity, the presence of local distortions, the rearrangement of valence charges due to dangling bonds and surfactants are all effects that influence XANES spectral shape. Although in bulk materials the signal from the surface represents a negligible contribution, the surface‐to‐volume ratio of NPs increases steeply with decreasing size, and the surface atoms in spherical NPs below 5 nm are already a few tens percent of the total amount. XANES provides valuable information on the local structure of surface atoms in such systems. Application of the high‐energy‐resolution fluorescence detected (HERFD) mode enhances the sensitivity of XANES. The reduced core–hole lifetime broadening allows the detection of smaller spectral changes and of features that would otherwise be invisible in conventional XANES.[^17^, ^47^, ^48^, ^49^] The application of HERFD to M_4,5_ edges of actinide materials has revolutionised the use of XANES in the field because it provides a direct probe of the 5f states with sufficient resolution to determine the oxidation state and observe the splitting due to f electron interactions.[^47^, ^49^, ^50^, ^51^] Although M_4,5_ HERFD XANES is considerably exploited in the field of actinides,[^15^, ^16^, ^52^] in only a very few cases has it been applied to NPs, and to the best of our knowledge no size effect has been reported yet at these absorption edges.

Here, we demonstrate the fundamental structural insight given by HEXS and HERFD XANES applied to ThO_2_ NPs synthesised by chemical precipitation followed by thermal treatment. By using HEXS, we were able to distinguish and quantify particles of different sizes by carefully analysing model structures of NPs, and, in particular, detect the presence of small clusters of atoms in the first stages of synthesis. HERFD XANES spectra at the thorium M_4_ edge for different steps of the synthesis show modifications of the f density of states (DOS), which, owing to the structural insight obtained by HEXS and theoretical simulations, we could correlate with the break of thorium local symmetry, most likely happening at the surface. The combination of these two techniques thus gives a complete overview of the structures of the NPs over all relevant length scales and can tackle the structural characterisation of non‐homogeneous samples of actinide NPs synthesised by chemical precipitation.

## Results and Discussion

Samples of ThO_2_ NPs were synthesised by sequential heat treatment of freshly precipitated Th^IV^ samples. Samples 1 and 2 result from drying in air the precipitate at 40 and 150 °C, respectively. To obtain ThO_2_ NPs of various sizes, freshly precipitated Th^IV^ was annealed at 400, 800 and 1200 °C in air in a muffle furnace. According to the PXRD analysis, samples 1 and 2 contain crystalline ThO_2_ NPs with average coherent scattering domains of 2.0 and 3.8 nm, respectively (see Figure S1 in the Supporting Information). Upon annealing, the particles grew significantly. The average sizes of the crystallites in the samples annealed at 400, 800 and 1200 °C were around 6, 34 and >100 nm, respectively. A summary of the sample size data obtained by PXRD and HRTEM can be found in Table S1, and for a detailed description the reader is referred to the report by Plakhova et al.^[9]^

**Figure 1:**
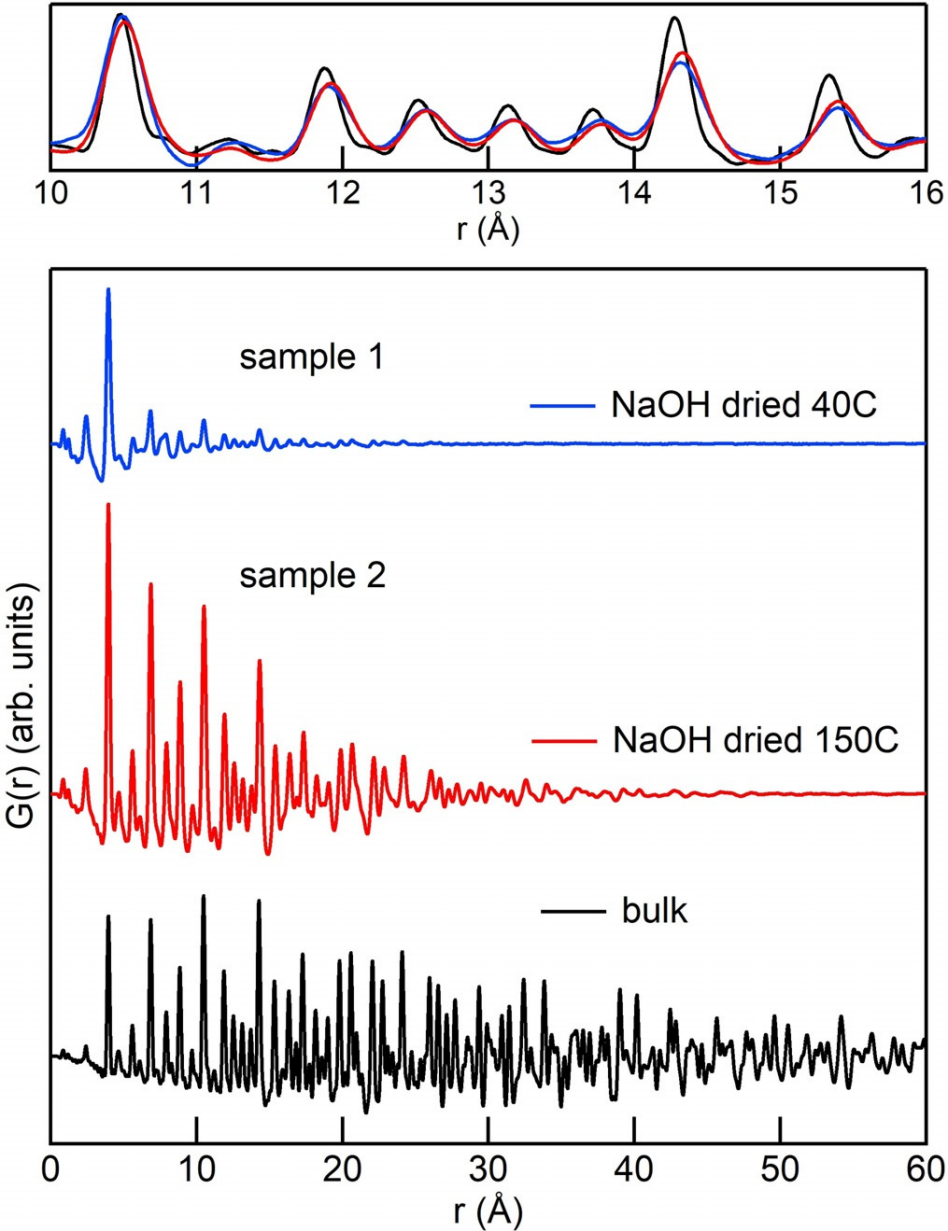
Bottom panel: unscaled PDFs for samples 1 and 2 and bulk ThO_2_. Top panel: the same PDF data, scaled and superimposed, are shown in the 10–16 Å range to highlight the shift to higher *r* of the peaks of samples 1 and 2.

Figure 1 shows the PDFs obtained by HEXS measurements on samples 1 and 2 and bulk ThO_2_. All the peaks in the PDFs of the samples correspond to the peaks of bulk ThO_2_, with the only exception of a feature of sample 1 at around 7.5 Å that will be discussed later. Due to the low scattering power of oxygen compared with thorium, the signal is dominated by Th–Th and Th–O pairs. The latter appear as distinct peaks below 7 Å, then above 7 Å the intensity drops rapidly and they become small shoulders at the bottom of the Th–Th peaks. Figure S2 in the Supporting Information shows the peak assignment based on the thorium‐centred distances in the ThO_2_ structure. Compared with bulk ThO_2_, the signals from the samples are progressively damped with increasing *r* and show only moderate broadening, a direct indication of the presence of NPs. The maximum distance at which oscillations are visible, that is, 4 nm for sample 1 and 6 nm for sample 2, marks the upper limit of NP size. Further inspection of the data also reveals that the peaks of samples 1 and 2 tend to be shifted to higher *r* compared with bulk ThO_2_. This is highlighted in the upper panel of Figure 1, in which data in the range 10–16 Å are superimposed and scaled.

Figure S3 in the Supporting Information shows the relative shifts of the peaks of the bulk and samples 1 and 2 in the range 0–20 Å. Sample 2 shows a linearly increasing shift to higher *r*, which indicates lattice expansion,^[40]^ in agreement with a recent report by Plakhova et al. based on PXRD measurements.^[9]^ The behaviour of sample 1 is more complex, with specific Th–Th peaks showing bigger deviations than the rest. Finally, we note that sample 1 presents an abrupt drop in intensity after the second peak, corresponding to the first Th–Th distance, and remains low for the rest of the signals. This is not the case for sample 2, for which the peak intensities decrease smoothly with increasing *r*.

**Figure 2:**
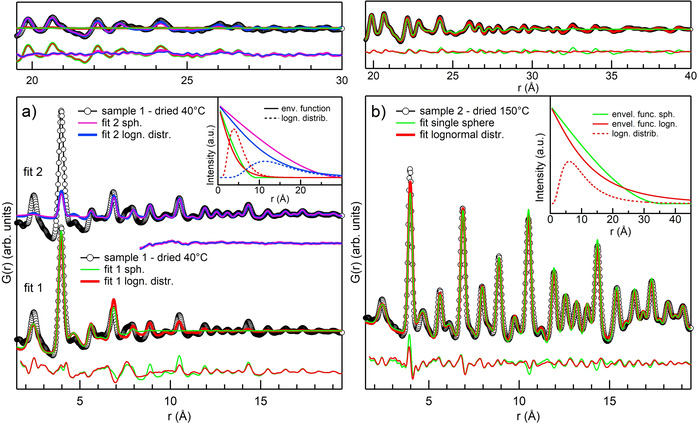
a) Data for sample 1 (black circles) and fit results (coloured lines). Fit 1 is on the full range of distances (1.5–30 Å), fit 2 on a reduced range (8.3–30 Å) but the fit results have been extrapolated to 1.5 Å. Residuals are shown below the relative fits and have not been extrapolated beyond the fit range. The inset shows the envelope functions (continuous lines) for all the fits together with the lognormal distributions (dashed curves). b) Data for sample 2 (black circles) and fit results with the single sphere (green) and lognormal (red) models. The inset shows envelope functions (continuous lines) for both fits and the resulting lognormal distribution (dashed line). The top panels of (a) and (b) show the high *r* range.

To extract quantitative information about the size and distribution of NPs, we first fitted the data with two semi‐empirical models based on imposing a size envelope function to the PDF of bulk ThO_2_: the single sphere model, which considers the sample as an ensemble of identical spherical NPs, and the lognormal distribution of spherical NPs model. The parameters for the fits were a scale factor, the lattice parameter *a* and the isotropic displacement parameters (*U*_iso_) for thorium and oxygen. In addition, the single sphere model fits the average diameter of the NPs (*P*_size_) and the lognormal model the mean diameter (*P*_size_) and the variance (*P*_sig_^2^) of the distribution. The parameters resulting from the fits together with the square of the residual (*R*_w_) are reported in Table 1, and the fits and corresponding data are compared in Figure 2 a,b. For sample 2 (Figure 2 b), both models give good fits. The average NP size from the single sphere model is 3.6 nm, whereas the resulting lognormal distribution spreads over a wide range of sizes and is characterised by a mean size of only 0.8 nm and a variance of 0.6 nm. The latter is shown in the inset of Figure 2 b, together with the envelope functions used by the semi‐empirical models to modulate the signal of the bulk. Comparison of the envelope functions and visual inspection of the fits show that with the single sphere model, the signal above the average diameter, that is, 3.6 nm, is set to zero, whereas with a lognormal distribution small oscillations are also found at high *r*. Indeed, the fit with the lognormal model has a slightly lower *R*_w_, reflecting the better agreement with data at both low and high *r*.

**Table 1:** Fit results obtained with the semi‐empirical models.^[a]^

	Model	Scale	*a* [Å]	Th *U*_iso_ [Å^2^]	O *U*_iso_ [Å^2^]	*P*_size_ [nm]	*P*_sig_^2^ [nm]	*R*_w_
sample 1	1 sph	1.49	5.610	0.011	0.075	0.93	–	0.30
	1 logn	1.80	5.607	0.010	0.080	0.5	0.2	0.28
	2 sph	0.13	5.616	0.009	0.293	3.1	–	0.29
	2 logn	0.18	5.616	0.008	0.060	1.5	0.7	0.31
sample 2	sph	0.96	5.619	0.008	0.059	3.6	–	0.17
	logn	1.13	5.619	0.008	0.063	0.8	0.6	0.12
bulk	–	0.57	5.600	0.004	0.036	–	–	0.11

[a] *P*_size_ is the NP size from the spherical model or the mean value of the lognormal distribution. *P*_sig_^2^ is the variance of the lognormal distribution.

The results for sample 1 are shown in Figure 2 a. Fitting sample 1 was more complex and we tried two *r* ranges: 1.5–30 Å (fit 1) and 8.3–30 Å (fit 2). Fit 1 in the full range (Figure 2 a, bottom) gives poor agreement above 10 Å: both models minimise the residual at low *r*, where the signal is stronger, and they are almost featureless above 10 Å.

By fitting over the full range of *r*, the single sphere model gives NPs with an average size of 0.93 nm and the lognormal model a very sharp distribution (*P*_sig_^2^=0.2 nm) peaking below 1 nm (*P*_size_=0.5 nm). *R*_w_ indicates that the lognormal fit 1 is slightly better. By excluding the low *r* from the fit range as in fit 2 (Figure 2 a, top), the agreement above 10 Å improves considerably. Above 10 Å, the sample is well represented by uniform spheres of 3.1 nm or by a lognormal distribution peak at 1.5 nm with a 0.7 nm variance. In contrast, when extrapolated to low *r*, fit 2 severely underestimates the signal below 5 Å. *R_w_* of fit 2, which cannot be compared with the others because of the different range, is slightly better for the single sphere model. However, visual inspection of the residuals in Figure 2 a shows that fit 2 is of identical quality for the purposes of this work.

The results for sample 1 are very interesting because they indicate that the data are not well described by a single distribution or a single NP size. One characteristic size dominates the signal at low *r* and is detected by fits in which the whole range is considered (fit 1). In this case, both models find average sizes below 1 nm. The residual at higher *r* definitely indicates the presence of larger particles that can only be fitted by excluding the signal at low *r*, as done for fit 2. This is not the case for sample 2, for which a single distribution is sufficient to reproduce the data. The semi‐empirical models provide a valuable insight into the different sizes present in the samples. However, they find considerable concentrations of NPs with diameters in the range 0.5–1.5 nm, predicted by assuming that diameters can take any value. This assumption is approximate below 1.5 nm and becomes appropriate only at larger diameters.

By cutting the smallest units with almost spherical shape out of a chunk of ThO_2_ and labelling each one with the larger Th–Th distance, only a few values between 0.5 and 1.5 nm were obtained. This is shown in Figure S4 in the Supporting Information. For a more precise identification of the NPs <1.5 nm in our samples, we implemented a fit model based on a minimal set of ThO_2_ NP structures cut from the bulk. Fits were carried out with diffpy‐CMI^[53]^ using the Debye equation.

**Figure 3:**
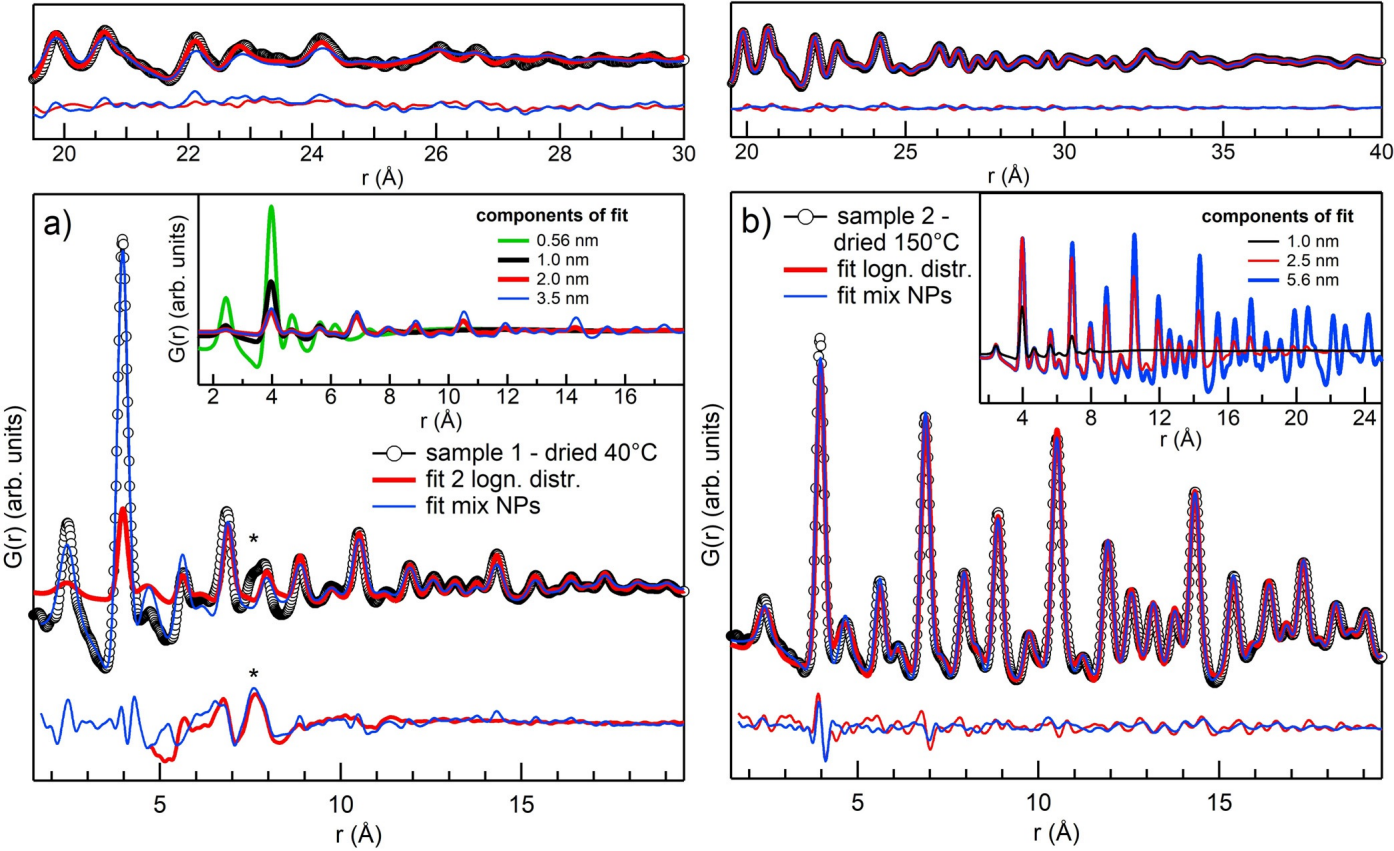
a) Data for sample 1 (black circles) compared with the fit results for the lognormal fit 2 (red line) and the fit with NP structures (blue line). b) Data for sample 2 (black circles) compared with the fit results for the lognormal fit (red line) and the fit with NPs structures (blue line). Residuals are shown below the fits and the insets of (a) and (b) show the calculated PDF for each NP structure added in the fit. The high *r* range is shown in the upper panels.

We isolated from bulk ThO_2_ a set of NPs of almost spherical shape with diameters between 0.5 and 6.0 nm. We carefully cut all structures below 1.5 nm while above 1.5 nm we constructed spheres centred on thorium with increasing radius up to 5.6 nm. The list of structures considered is reported in the Supporting Information. We fitted samples 1 and 2 in the ranges 1.7–40 and 1.7–60 Å, respectively, with the minimal subset of structures. The PDFs of ideal structures were calculated from the atomic coordinates using the Debye scattering equation implemented in diffpy‐CMI (DebyePDFGenerator and DebyePDFCalculator). Each NP structure adds two parameters to the fit: a lattice expansion coefficient and a scale factor. The latter, when divided by the sum of all the scale factors, gives the concentration of the corresponding structure in the sample. The isotropic displacement parameters (*U*_iso_) for thorium and oxygen common to all structures were also fitted. To find the best fit, we first added big NPs and optimised the agreement in the tail of the PDF signal, where oscillations are weak and only the largest NPs contribute. Extrapolating the fit to lower *r* and comparing it with the data reveals where the intensity is still missing and allows an estimation of the sizes that should be included in the ensemble to improve the fit. Due to the limited number of structures and samples, we proceeded with a manual fit that allows visual inspection of the results. The results of the new fits are reported in Table 2. Figure 3 a,b present the new results in comparison with those of the lognormal fits from semi‐empirical models. The new fits improve the agreement for both samples. For sample 2, *R*_w_ decreases slightly and inspection of the residuals in Figure 3 b reveals small improvements over the whole *r* range. For sample 1, the *R*_w_ improves considerably compared with that of lognormal fit 1, which was over the full range. *R*_w_ of lognormal fit 2 cannot be compared because a different range was used. However, the direct comparison shown in Figure 3 a illustrates that above 6 Å the fits give very similar results, whereas below 6 Å the new fit reproduces very well the abrupt decrease in intensity. According to the results reported in Table 2, sample 1 comprises 61.3 % of 0.56 nm NPs, that is, the smallest units that can be cut from bulk ThO_2_, mixed with 24.5 % of 1.0 nm NPs and low concentrations of 2.0 and 3.5 nm NPs. Sample 2 consists of a more homogeneous mixture of 1.0 nm (24 %), 2.5 nm (41 %) and 5.6 nm (34 %) NPs.

**Table 2:** Fit results for samples 1 and 2 with the set of NP structures.

NP	Scale	Conc. [%]	a*exp. coeff.	Th *U*_iso_ [Å^2^]	O *U*_iso_ [Å^2^]	*R*_w_
*sample 1, dried at 40 °C*						0.21
0.56 nm	0.60	61.3	5.614	0.0133	0.0467	
1.0 nm	0.24	24.5	5.580			
2.0 nm	0.072	7.4	5.601			
3.5 nm	0.067	6.8	5.619			
*sample 2, dried at 150 °C*						0.09
1.0 nm	0.24	24	5.603			
2.5 nm	0.41	41	5.603	0.0075	0.0075	
5.6 nm	0.34	34	5.624

The calculated PDFs of each NP contributing to the fits are shown in the insets of Figure 3 a,b. These results confirm what the semi‐empirical models suggested: sample 1 has a high concentration of very small particles mixed with larger ones, whereas sample 2 is described by a more homogeneous distribution of sizes. Notably, the high concentration of 0.56 nm units disappears upon heating at 150 °C. This small octahedral unit that we artificially cut from ThO_2_ bulk is very similar to thorium hexamer clusters, which have been frequently reported in literature.[^18^, ^19^, ^20^, ^21^, ^44^] We notice that even if we applied a fit exclusively based on NP structures, a fit approach mixing NP structures with small sizes and a lognormal distribution gives results of very similar quality. These two approaches are compared in Figure S5 and Table S2 in Supporting Information for sample 1. Nevertheless, with the fit using only NP structures, a lattice parameter for each structure can be fitted and different NP shapes could be easily implemented as well as core–shell structures. The high flexibility of this method may help to achieve even more detailed structural information, which could be exploited in studies on larger data sets.

Despite the good results obtained, there is still room for substantial improvement, especially for sample 1. The main contribution to the residual is the peak at 7.6 Å, indicated with an asterisk in Figure 3 a, which does not belong to fluorite ThO_2_. A similar feature was previously reported by Magini et al.^[31]^ in their investigation of hydrolysed thorium salts. They assigned it to aggregates of oxygen‐centred tetrahedra interconnected through facet‐sharing at an early stage of the synthesis of ThO_2_. In our case, the peak could be the result of surface disorder on the 1.0 nm NPs. This disorder could cause a splitting of the peak at 7.75 Å, and would correspond to thorium at the surface. The assignment of this peak, as well as a deeper insight into the early stages of Th^IV^ hydrolysis, requires the collection of a bigger set of data, which will be the focus of future investigations.

**Figure 4:**
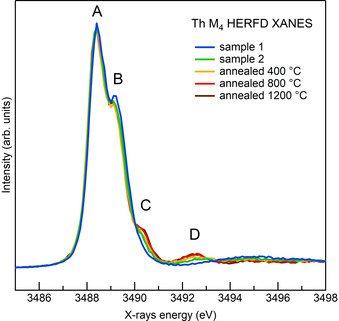
Thorium M_4_ edge HERFD data for samples 1 and 2 as well as the samples annealed at 400, 800 and 1200 °C. The data are normalised to the total spectral area.

Figure 4 shows the thorium M_4_ edge HERFD spectra recorded for samples 1 and 2 and for samples annealed at 400, 800 and 1200 °C. XANES analysis at the M_4_ edge of actinides corresponds to the excitation of an electron from the 3d_3/2_ to the 5f_5/2_ state and provides direct access to the f‐DOS of the actinide. The spectrum of the sample annealed at 1200 °C is identical to that of bulk ThO_2_ and presents four main features: the main peak A at the absorption edge, two shoulders labelled B and C, and feature D well separated from the absorption edge region. Features C and D are absent in sample 1, only slightly visible in sample 2 and they progressively grow for the NPs annealed at high temperatures. We also note that feature B is slightly more intense in sample 1. However, the difference is very small and cannot be associated with the trend observed for features C and D. Further investigation of larger sets of samples is needed to confirm the effect on feature B.

The progressive growth of features C and D in the thorium M_4_ edge HERFD data seems to follow the increase in crystallinity and size of NPs. The results of the PDF fitting indicate that the precipitate dried at 40 °C (sample 1) predominantly comprises small units similar to thorium hexamers and 1.0 nm NPs. Drying the precipitate at higher temperature (sample 2) stimulates the growth of the existing NPs and causes the loss of the smaller units detected by the PDF fitting. From the PXRD data, we know that the growth continues with annealing at progressively higher temperature in the range 400–1200 °C. The sensitivity of the thorium M_4_ edge to crystallinity and NP size is quite a novelty and may sound surprising. Indeed, 5f states are generally considered strongly localised, not involved in chemical bonding and only mildly sensitive to the crystal field of neighbouring atoms. The above observations fit better the 4f states of lanthanides rather than the 5f states of the early actinides. The latter are spatially more extended, more sensitive to the presence of neighbouring atoms and more prone to participate in bonds. The case of actinyl ions, in which the actinide forms two very short and strong linear bonds with oxygen atoms, is a well‐known case of chemical bonding involving 5f orbitals.^[54]^

To assess whether the observed effects correlate with size reduction, we need to understand the nature of each feature in the spectrum and rationalise whether the disappearance of peaks C and D is compatible with this hypothesis. In the absence of a large set of well‐characterised references, simulations are the only way to shed light on the nature of spectral features. Butorin et al.^[55]^ recently modelled the M_4_ HERFD spectra of ThO_2_ within the single impurity Anderson model, which fully accounts for electron correlations and treats interatomic interactions as a perturbation. Features A and B were well reproduced by the *O_h_* crystal‐field effect on the 5f orbitals of thorium, whereas feature D is obtained by adding ligand‐to‐metal charge transfer driven by Th 6d–Th 5f–O 2p hybridisation. Even if some multiplet poles arise corresponding to feature C, they are too weak to generate a shoulder and the assignment of feature C remains open. In the approach used by the authors, based on atomic physics, it is difficult to implement effects due to size and complex local distortions because the influence of neighbouring atoms is included as a perturbation, the strength of which is regulated by empirical parameters. The number of parameters increases very quickly with the lowering of local symmetry, as expected for surface atoms or small clusters. Approaches that naturally account for the surrounding atoms, like those based on DFT, have the advantage of avoiding empirical parameters to account for local symmetry and the redistribution of valence charge. These approaches only partially include electron correlations, making them unsuited to treating strongly correlated f electron systems. Indeed, this theory fails to reproduce the M_4_,_5_ edges of 4f elements (lanthanides). The 5f states are less localised and the calculations performed following this scheme are less questionable if the purpose is to reproduce observed trends and to deduce valuable information. Moreover, for actinide materials such as ThO_2_, uranyl‐type or U^VI^ compounds, which have empty 5f orbitals in the ground state, the DFT approaches are well‐suited. To the best of our knowledge, only a few attempts have been made to simulate XANES spectra at the M_4_,_5_ edges of early actinides using DFT‐based codes.[^56^, ^57^, ^58^] The outcomes were promising, even if the implications of disregarding electron correlations in systems with f electrons have not been discussed explicitly and a comparative study of M_4_,_5_ edges of early actinides simulated by using the two approaches is still missing.

**Figure 5:**
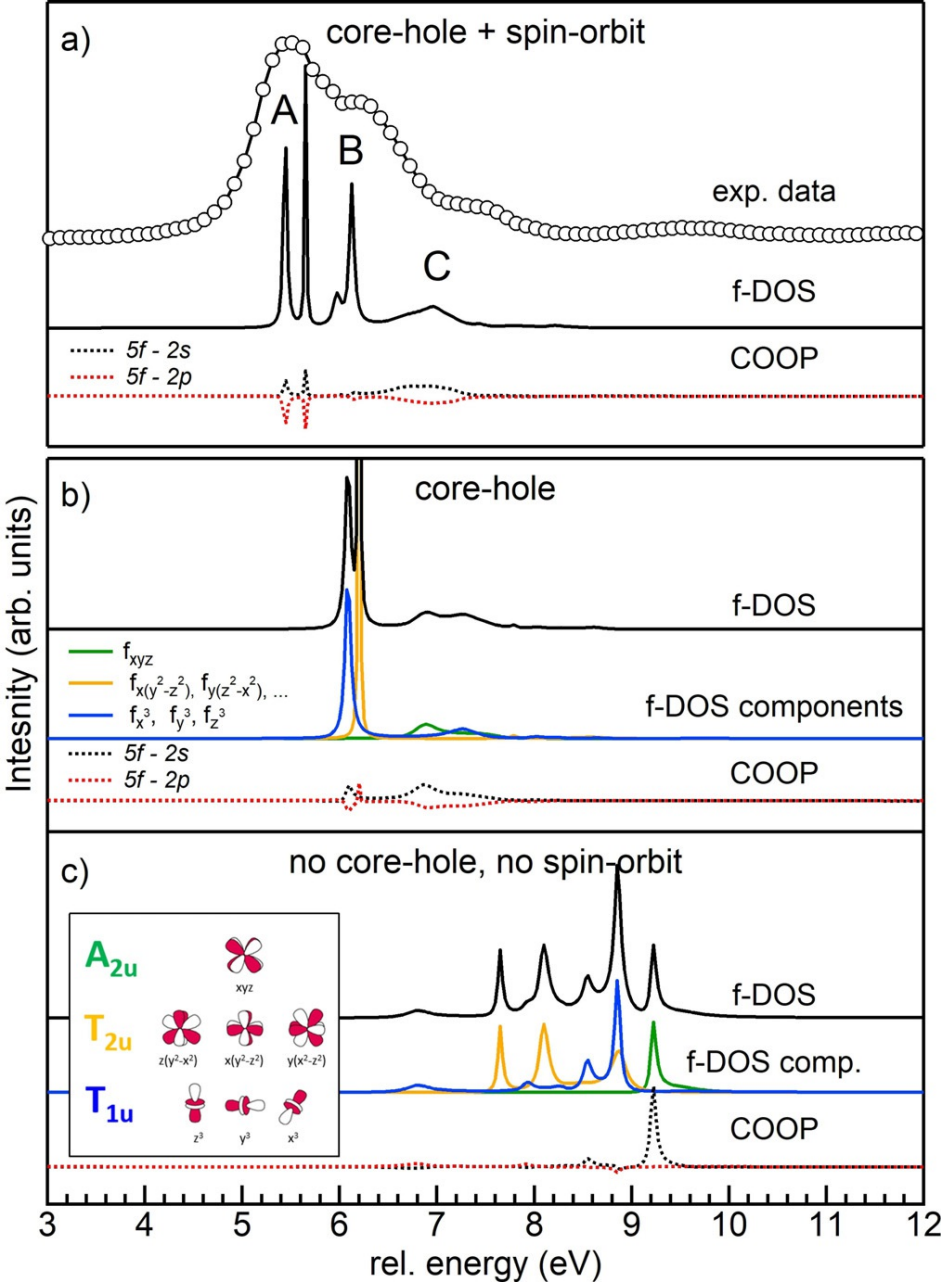
FDMNES simulations of bulk ThO_2_ and compared with experimental HERFD data on the sample annealed at 1200 °C (panel a, black circles). a) Simulations obtained with core–hole and spin–orbit effects included: the total f‐DOS (black line) and the COOPs of Th 5f with O 2s (black dotted line) and 2p (red dotted line). b) Results obtained without the spin–orbit effect. c) Results obtained without the spin–orbit and core–hole effects. In (b) and (c), the total f‐DOS and COOPs are indicated by the same colour code as in (a). In addition, in (b) and (c) the decomposition of the f‐DOS into the cubic set is shown (A_2u_ in green, T_2u_ in yellow and T_1u_ in blue). A sketch of the f‐orbital cubic set in *O_h_* symmetry is drawn in (c).

We simulated the f‐DOS and M_4_ edge XANES spectra of bulk ThO_2_ by using FDMNES[^59^, ^60^] to elucidate the nature of the spectral features. Figure 5 shows the f‐DOS data with both core–hole and spin–orbit effects included (Figure 5 a), with only the core–hole (Figure 5 b) and without both (Figure 5 c). Each panel reports the total f‐DOS (black curve) and the crystal overlap orbital populations (COOPs) between the 5f orbitals of thorium and the 2s (black dotted line) and 2p (red dotted line) orbitals of neighbouring oxygen. The COOPs quantify the covalency of the Th−O bond by integrating the product of their atomic orbitals inside a sphere centred on the bond axis.^[61]^ Positive/negative COOPs indicate bonding/anti‐bonding character. Figure 5 b,c also shows the decomposition of the total f‐DOS into the cubic set of f orbitals, which in *O_h_* symmetry splits into three groups, sketched in Figure 5 c. By comparing Figure 5 b and c, we first notice that the core–hole effect has a strong impact on the f‐DOS. It lowers its energy and increases its sharpness and intensity close to the edge. Comparison of Figure 5 a and b shows that the effect of the spin–orbit interaction is to introduce additional splitting to that originating from the local geometry. The final f‐DOS resulting from the inclusion of the core–hole and spin–orbit effects (Figure 5 a, black curve) shows three groups of features, the energy separation and relative intensity of which are in good agreement with the experimental data. For further insight into the nature of each group of features, we look at the projection of the total f‐DOS into the cubic set for simulations without the spin–orbit effect (Figure 5 b,c). ThO_2_ crystallises in the fluorite structure (*Fm*‐3*m* space group), with thorium having eight neighbouring oxygen atoms located in the vertices of a regular cube. Under this local symmetry, the f orbitals expressed in the cubic set split into three groups: T_1u_ (f${}_{x{}^{3}}$ , f${}_{y{}^{3}}$ , f${}_{z{}^{3}}$ ), T_2u_ (f${}_{x(y{}^{2}-z{}^{2})}$ , f${}_{y(z{}^{2}-x{}^{2})}$ , f${}_{z(x{}^{2}-y{}^{2})}$ and A_2u_ (f_*xyz*_). This is indeed what we observe in Figure 5 b,c. The ordering in the absence of a core–hole effect is in agreement with expectation and with previously published results for UO_2_:^[62]^ the contribution of lowest energy shows T_1u_ symmetry (small blue peak at ca. 6.8 eV) and the single orbital A_2u_ (f_*xyz*_) is the most destabilised and highest in energy. Indeed, f_*xyz*_ is directed towards the vertices of the cube of oxygen atoms and interacts more strongly with them. This is also seen in the COOPs, which show a strong bonding interaction of O 2s and U 5f at A_2u_. It is interesting to note that the core–hole effect (Figure 5 b) lowers the energies of all orbitals and confers on them the extreme sharpness and high intensity of T_1u_ and T_2u_, which suggests an increased localisation of these orbitals in the presence of the core–hole potential, whereas A_2u_ remains isolated at higher energy and is broadened. The bonding character of Th 5f A_2u_–O 2s is maintained with the addition of a significant anti‐bonding interaction with O 2p.

**Figure 6:**
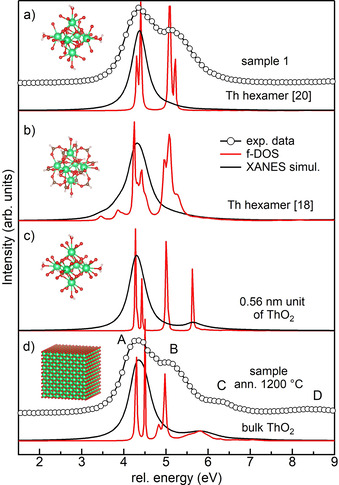
Simulations of the total f‐DOSs (black lines) and XANES spectra (red lines) for thorium hexamers from ref. [20] (a) and ref. [18] (b), the 0.56 nm unit of ThO_2_ (c) and bulk ThO_2_ (d). The experimental HERFD data for sample 1 and the sample annealed at 1200 °C are shown above the simulations in (a) and (d), respectively. Each panel shows a scheme of the simulated structure.

Unfortunately, we cannot project the f‐DOS in Figure 5 a, obtained considering the spin–orbit effect, on a basis set symmetrised for the *O_h_* double group. However, we know from group theory that T_1u_ and T_2u_ are further split into two subgroups and A_2u_ remains unaffected. The parallel with the results presented in Figure 5 b suggests the assignment of peaks A and B of the experimental M_4_ HERFD to f orbitals with T_1u_ and T_2u_ symmetry and peak C to the single A_2u_ orbital. The COOPs shown in Figure 5 a are also very similar to those of Figure 5 b and further support our assignment. Despite the observation that the f‐DOSs calculated by FDMNES are in good agreement with experimental data, the simulated XANES (see Figure 6 d) calculated once the spin–orbit effect is added completely disregards part of the f‐DOS and peak B is not reproduced. The difference between the f‐DOS and simulated XANES stems from the selection rules, which allow transitions only to states with *j*=5/2 for M_4_, whereas transitions to both *j*=5/2 and *j*=7/2 states are allowed for M_5_.^[63]^ Figure S6 in the Supporting Information allows comparison of the simulations of M_4_ and M_5_. The collection of M_5_ data would help clarify the absence of peak B in the simulations. Unfortunately, the spectrometer used in this experiment, a Johan‐type spectrometer operating at 65–90°, does not supply the emission energy needed for thorium M_5_ HERFD. Measurements of thorium M_4_ spectra are only feasible with spectrometers having a different design.^[64]^

The total f‐DOS splitting grasps the physics behind spectral features, because the energy separation and relative intensity of the f‐DOS agree fairly well with experimental data. We can therefore conclude that peaks A, B and C arise from the combined splitting of the crystal field and the spin–orbit effect on thorium 5f orbitals. Peak C, in particular, is the most affected by the interaction with oxygen nearest neighbours. As expected, we do not reproduce peak D, which arises from ligand‐to‐metal charge transfer,^[55]^ an effect that the current approach disregards.

We know from the PDF that the principal constituents of sample 1 are small units similar to thorium hexamer clusters and ThO_2_ NPs of 1.0 nm. Both structures contain only a few thorium atoms (6 and 13), which are all, except for the central thorium in the 1.0 nm NP, on the surface and most probably experience a variation of their local environment. The disappearance of peak C in the XANES spectrum of sample 1 may therefore reflect this change. To confirm this hypothesis, we simulated the thorium M_4_ XANES spectra of two thorium hexamers, the structures of which are reported in the literature,[^18^, ^20^] and of the 0.56 nm ThO_2_ NP that we used in PDF fitting. Both thorium hexamers comprise six thorium atoms linked together as in ThO_2_. We added six H_2_O molecules to the 0.56 nm unit, similarly to what is found in thorium hexamers. Figure 6 shows schematics of all the structures and a comparison of the f‐DOS and XANES simulations of the thorium clusters and bulk ThO_2_. All simulations were shifted in energy to have the first feature aligned. Interestingly, the total f‐DOS of the 0.56 nm NP maintains the three features observed in the bulk, whereas the thorium hexamers only have the first two intense features and have lost the last, corresponding to peak C. The XANES simulations give good agreement with the f‐DOS data, with the exception of the systematic absence of the second peak, which confirms that the selection rules are the origin of the discrepancy. Although the thorium hexamers (Figure 6 a,b) reproduce the observed disappearance of peak C, the very similar 0.56 nm NP does not (Figure 6 c). The reason for this is the orientation of the four external oxygen atoms bound to each thorium: in the 0.56 nm NP they sit at the corners of the cube centred on thorium, as in bulk ThO_2_. In thorium hexamers, the same oxygen atoms are rotated by approximately 45° around the axis connecting two opposite thorium. This rotation breaks the *O_h_* symmetry around thorium and stabilises more the anti‐bonding A_2u_ orbital, which reduces the superposition with oxygen. We additionally remark that the relative intensity of the f‐DOS peaks of the thorium hexamers differs from that of bulk ThO_2_. In particular, the features corresponding to peak B are more intense in the thorium hexamer, a hint that the relative intensity of peaks A and B may also be sensitive to changes in local environment and that the slight increase in intensity of peak B observed for sample 1 may be relevant.

**Figure 7:**
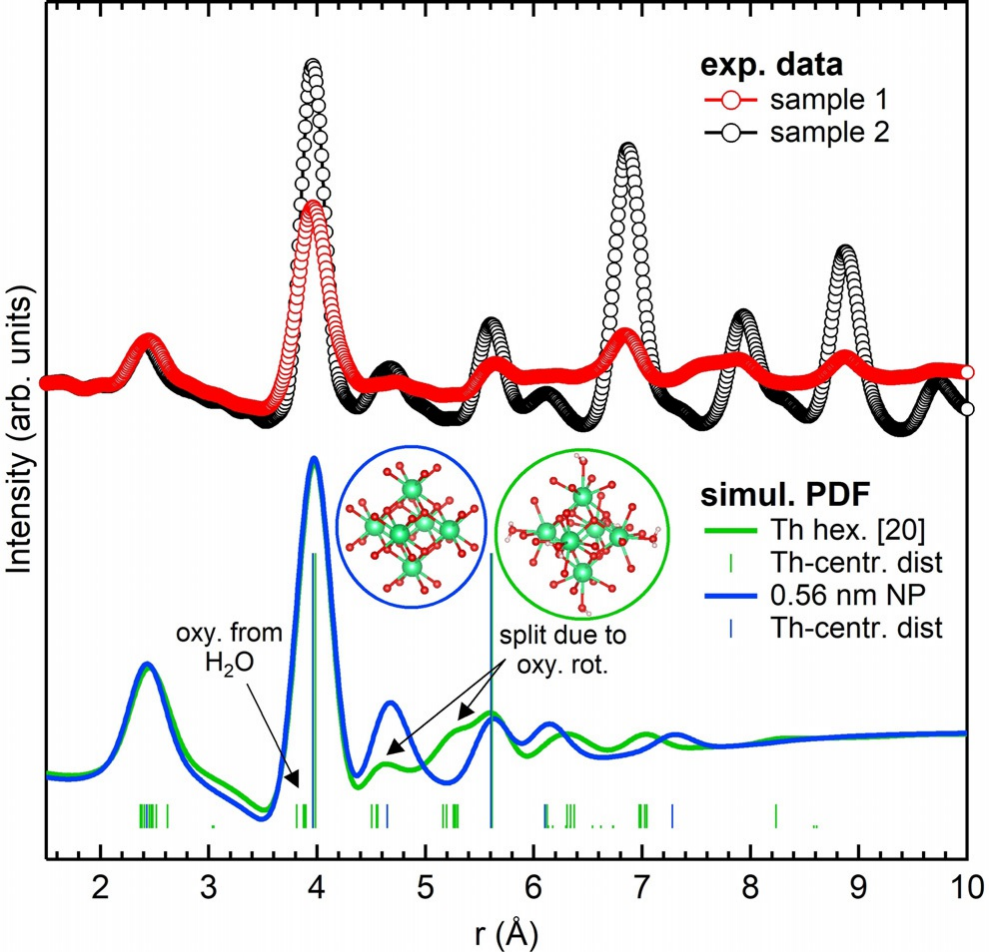
PDF experimental data for samples 1 (red circles) and 2 (black circles) are shown together with the calculated PDFs for the structure of the 0.56 nm NPs cut from bulk ThO_2_ (blue line) and thorium hexamer from ref. [20] (green line). The vertical sticks at the bottom indicate the thorium‐centred pair distances in the two structures. The heights of the sticks are proportional to the atomic number (Th=90, O=8, H=1) and not to the frequency of the represented distance.

HEXS and HERFD data, when thoroughly analysed, as we did in this work, have been demonstrated to be a very powerful tool for unravelling the complexity of ThO_2_ NP structures. The PDF reveals that samples are a mixture of particles of different sizes and that a large percentage of subnano NPs are initially formed, which then disappear to favour larger NPs when the Th^IV^ precipitate is dried at 150 °C. This finding suggests a formation mechanism in which small units, which we found to be similar to thorium hexamers, are incorporated into larger NPs or aggregate with each other. The strongest evidence for the presence of subnano units comes from the PDF sensitivity to both short‐ and medium‐range order: the decrease in intensity of Th–Th peaks above 5 Å can only be reproduced by units with only two Th–Th distances. We notice that at these distances the presence of additional thorium atoms, the positions of which deviate from those of crystalline ThO_2_, would still contribute to the PDF signal, albeit differently from what would happen for EXAFS. The latter has a higher resolution and is only sensitive to short‐range order. As a result, a spread of bond distances that does not suppress the coherence needed to observe PDF peaks is sufficient to flatten the signal from higher shells in EXAFS analyses. Figure 7 shows the simulated PDF signals for the 0.56 nm unit cut from ThO_2_ and for the thorium hexamer.^[20]^ At the bottom of Figure 7, all the thorium‐centred distances are represented as sticks and show the local disorder present in the thorium hexamer. Although for the thorium hexamer the spread of Th–O distances covers the range 2.37–2.62 Å, the first peak of the simulated PDF is not affected dramatically, whereas a similar spread has a strong impact on EXAFS, as reported by Rothe et al.^[28]^ The scarce structural information available on amorphous hydrous ThO_2_ indeed comes from EXAFS measurements and are limited to the first Th–O shell, which can only be fitted with multiple Th–O distances differing from those of crystalline ThO_2_.

The signals from higher shells are typically very low making it impossible to disentangle and quantify static disorder and coordination numbers. The sensitivity of HEXS to short‐ and medium‐range order allows the sample to be characterised on all the relevant length scales and reveals the presence of subnano units mixed with larger NPs. NPs below 1.5 nm have significant local disorder, but they still preserve the ThO_2_ structure at short‐ and medium‐range. The results of this study suggest that subnano units similar to thorium hexamer clusters rather than an amorphous phase are intermediates in the formation of ThO_2_ NPs. This picture is in line with the findings of Hu et al.^[44]^ and with the more general understanding emerging that the hydrolysis of tetravalent cations can result in well‐defined metal oxide/hydroxide aggregates.[^11^, ^23^]

In contrast to HEXS, XANES is sensitive to the short‐range order and to the stereochemistry around the absorber. More detail about the local coordination of the absorber can be extracted. The complexity of XANES analysis is often a bottleneck to extracting information. However, the theoretical simulations available today can greatly help their interpretation, and knowledge of the structure of the sample can guide the attempts to theoretically reproduce the spectral changes. In this regard, the coupling of HEXS and XANES provides more structural information and when applied to small objects can be an invaluable tool to investigate surface modifications. In this study, we have demonstrated that the trend observed in thorium M_4_ HERFD XANES can be rationalised by a change in the local symmetry around the thorium atoms to that found in thorium hexamer clusters, with the rupture of *O_h_* symmetry caused by the rotation of the oxygen ligands. Such a break in the local symmetry is expected to affect in particular surface atoms, with dangling bonds leaving more freedom for rearrangement. The predominance of surface atoms in sample 1, mainly constituted by NPs <1.5 nm, results in the disappearance of features C and D of the M_4_ HERFD, which are specifically sensitive to oxygen neighbours and Th 6d–O 2p hybridisation,^[55]^ respectively. Compared with our previous results on analogous samples,[^9^, ^17^] this investigation determines with more accuracy the characteristic sizes composing the samples. In particular, the presence of NPs <1.5 nm is in agreement with the very low Th–Th coordination number found by EXAFS and with the effect previously observed in thorium L_3_ edge XANES and ascribed to low‐coordinated thorium atoms at the surface as well as disorder at the surface.^[17]^

By illustrating the opportunities of using HEXS and HERFD XANES to investigate ThO_2_ NPs, our work opens the way to a thorough investigation of the mechanism of NP formation for ThO_2_ and more generally for actinide oxides. The effects of the initial chemical conditions, polymerisation of the initial Th^IV^ precipitates and the stability of the NPs over time can be addressed with this methodology. The latter is of particular interest because ThO_2_ NPs, in contrast to NPs of other actinide dioxides synthesised by the same chemistry route, show an increase in size and crystallinity over time (ageing effect) depending on the conditions. Our findings on ThO_2_, in comparison with recent results on CeO_2_ ^[65]^ and PuO_2_[^15^, ^16^] NPs synthesised under similar conditions, show differences in systems that are often indicated as very similar. The fact that thorium has only one oxidation state, unlike cerium and plutonium, prevents the accommodation of charge imbalance with oxidation or reduction at selected sites. This limitation may be compensated by increased disorder.[^9^, ^14^, ^17^] Comparative studies of the structural properties of AnO_2_ NPs could help to assess the similarities and differences, and shed light on the different nucleation mechanisms. Altogether, the data and analysis presented here demonstrate that investigating very small NPs with HEXS and HERFD provides important insights into the structure of NPs and the local environment at their surface. Combining the two techniques is particularly important when samples are far from ideal, that is, without very narrow size distribution and a uniform shape. Being able to extract structural information on such samples is of paramount importance, because it sheds light on the mechanisms of NP formation that may be acting in the environment.

## Conclusions

We have investigated the structures of ThO_2_ NPs in two samples representing the initial and a more advanced step of their synthesis by chemical precipitation by combining HEXS and HERFD XANES at the thorium M_4_ edge. The analysis of the PDF by semi‐empirical methods and fits based on real NP structures revealed that the sample dried at mild temperature (40 °C, sample 1) contains mainly ThO_2_ NPs <1.5 nm. Drying at higher temperature (150 °C, sample 2) results in larger NPs and no subnano units. HEXS analysis revealed that the initial Th^IV^ precipitate contains a large number of seeds for ThO_2_ NPs. Moreover, increasing the drying temperature to 150 °C promoted recrystallisation to the more thermodynamically stable ThO_2_ phase. HERFD analysis at the thorium M_4_ edge unexpectedly showed a remarkable effect on two spectral features, that is, peaks C and D, which progressively grew with increasing crystallinity and particles size. FDMNES simulations shed light on the nature of feature C, revealing its marked sensitivity to the break of *O_h_* symmetry around the thorium ions, which, considering the morphologies of our samples, is most likely caused by a modified arrangement of oxygen ligands at the surface. Thorium M_4_ edge HERFD analysis demonstrated a high sensitivity to the local structure at the surface when applied to small NPs, information that is very difficult to obtain and of fundamental importance for understanding the interaction mechanisms of NPs. HEXS and HERFD supported by suitable theoretical simulations are therefore powerful tools for investigating actinide nanomaterials, especially when non‐homogeneous samples such as those obtained in solution chemistry are the focus of the investigation.

## Experimental Section

The ThO_2_ samples were synthesised by sequential heat treatment of freshly precipitated Th^IV^ samples. We first mixed aqueous solutions of 0.1 m thorium nitrate pentahydrate and 3 m sodium hydroxide, which resulted in the formation of a Th^IV^ precipitate. Portions of the freshly precipitated Th^IV^ sample were dried in air at 40 °C (sample 1) and 150 °C (sample 2). To obtain ThO_2_ nanoparticles of various sizes, the freshly precipitated Th^IV^ was annealed at 400, 800 and 1200 °C in air in a muffle furnace. A summary of synthesis procedures and structural characterisation by PXRD and HRTEM can be found in Table S1 and Figure S1 in the Supporting Information.

We collected the HEXS data for samples 1 and 2 at the ID15A^[66]^ beamline of the European Synchrotron Radiation Facility (ESRF, Grenoble). The data were collected at room temperature, the incident energy was set to 120 keV and we measured up to 30 Å^−1^ using a Dectris CdTe 2M pixel detector. The samples were sealed in kapton capillaries (double confinement) and the signal from an empty capillary was used for background subtraction. Patterns were corrected for detector geometry, response and transparency, and integrated by using a locally modified version of pyFAI^[67]^ with outlier filtering.

The PDF was calculated from the resulting powder diffraction patterns by using modules from PDFgetX3^[68]^ and the data were corrected for electronic noise and weak spurious signals by fitting the high‐angle part of the calculated *F*(*q*) to a weighted spline to remove outliers following a procedure similar to that described previously.^[69]^ The PDF Gaussian dampening envelope due to limited *Q*‐resolution and *Q*‐broadening was obtained from the fit of a reference sample and fixed at these values for the NPs. The maximum scattering vector **Q** of the data used for the generation of the PDF was 26 Å^−1^. We fitted the NP data with diffpy‐CMI^[53]^ using three different models: periodic models using a single spherical or lognormal distribution of nanoparticle sizes^[70]^ and a model based on a set of discrete NP structures. The number of refined parameters was kept as low as possible, and visual inspection and the residual *R*_w_ were used to evaluate the goodness of fit. The refined parameters for the first two models were the lattice parameter *a*, the *U*_iso_ of thorium and oxygen, the mean particle size (*P*_size_) and the variance of the lognormal distribution (*P*_sig_^2^). For the model implementing a set of NP structures, two parameters were common to all structures, the *U*_iso_ of thorium and oxygen, and two additional parameters were specific to each NP in the set, a scale factor and a lattice expansion parameter. To limit the number of parameters, *δ*_2_, which takes into account the first‐neighbour interaction, was fixed to 2.0 Å for all fits.

The XANES spectra of the thorium M_4_ edge were collected in the HERFD mode at the ID26 beamline^[71]^ of the ESRF. The incident energy was selected with a Si(111) double crystal monochromator. The rejection of higher harmonics was achieved with three silicon mirrors at angles of 3.0, 3.5 and 4.0 mrad relative to the incident beam. The X‐ray emission spectrometer[^72^, ^73^] was equipped with three Ge(220) spherically bent crystal analysers (1 m radius) at a Bragg angle of 80° to collect the maximum of the M_β_ emission line of thorium (ca. 3148.6 eV). The energy resolution estimated by the FWHM of the elastic peak was 0.4 eV. The samples were measured as dried powders sealed with double kapton confinement in the sample‐holder.

We simulated the thorium M_4_ edge XANES spectra by using the FDMNES code.[^59^, ^60^] The scattering potential around the thorium absorber was calculated self‐consistently within a radius of 5 Å. The best agreement was obtained with the inclusion of a fully screened core–hole and by using the finite difference method (FDM). Relativistic effects and spin–orbit interactions were included. An example of the input file used for the simulations is provided in the Supporting Information.

## Conflict of interest

The authors declare no conflict of interest.

## Supporting information

As a service to our authors and readers, this journal provides supporting information supplied by the authors. Such materials are peer reviewed and may be re‐organized for online delivery, but are not copy‐edited or typeset. Technical support issues arising from supporting information (other than missing files) should be addressed to the authors.

Supplementary
