# Supplementary material for: The Application of HEXS and HERFD XANES for Accurate Structural Characterisation of Actinide Nanomaterials: The Case of ThO2
Source: Chemistry. 2020 Nov 12;27(1):252–63. doi: 10.1002/chem.202003360 (PMC7839789; doi:10.1002/chem.202003360)
Supplement: Supplementary file 1 — Supplementary [file CHEM-27-252-s001.pdf]

Chemistry–A European Journal

Supporting Information

The Application of HEXS and HERFD XANES for Accurate Structural Characterisation of Actinide Nanomaterials: The Case of ThO_2

Lucia Amidani,*[a, b] Gavin B. M. Vaughan,[c] Tatiana V. Plakhova,[d] Anna Yu. Romanchuk,[d] Evgeny Gerber,[a, d] Roman Svetogorov,[e] Stephan Weiss,[b] Yves Joly,[f] Stepan N. Kalmykov,[d] and Kristina O. Kvashnina[a, b, d]

XRD and HRTEM characterization

X-ray diffraction (XRD) was used for preliminary characterization and for a first estimation of NP size. Laboratory measurements were done with a Bruker D8 Advance diffractometer with a Cu Kα radiation source (wavelength 1.54 Å). Synchrotron X-ray diffraction was collected at the Kurchatov Institute Synchrotron radiation source in Moscow with a MarCCD165 detector. Data were collected with monochromatic radiation of wavelength λ = 0.8 Å, focused on a spot of 400 µm. Microstructure evaluation was performed using JEOL-2100F high-resolution transmission electron microscope (HRTEMs) at an accelerating voltage of 200 kV. The XRD and HRTEM data for all studied samples is presented on Figure S1.

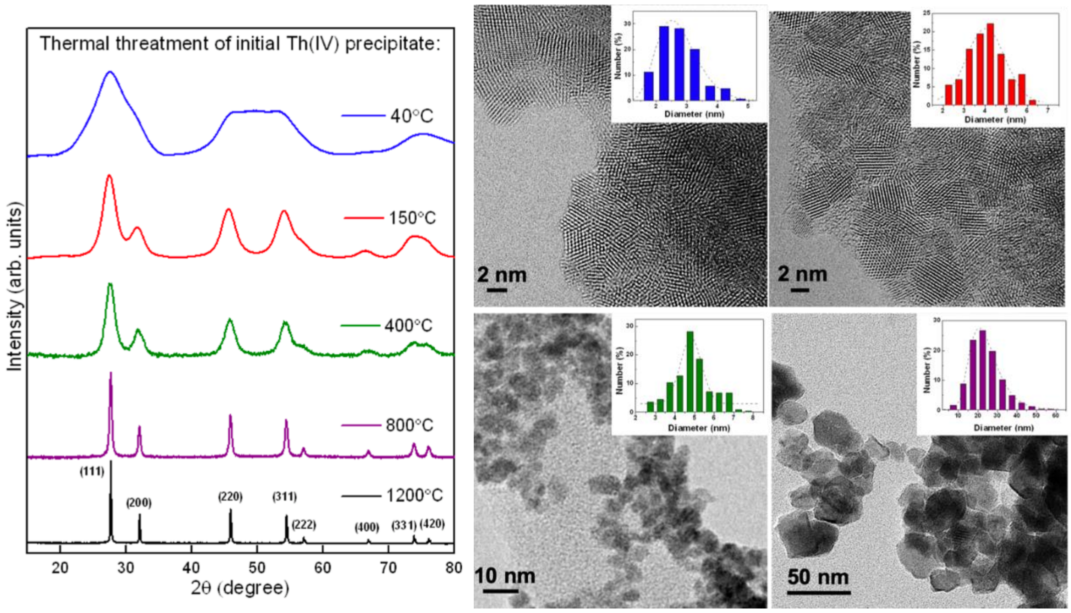

Figure S1: left: XRD patterns of ThO_2 samples synthesized from 0.1 M $Th(NO_3)_4$ and 3M NaOH with further drying at 40 °C (sample 1) and 150 °C (sample 2) in the air and samples obtained by annealing at 400 °C, 800 °C or 1200 °C.[1] Right: HRTEM images and particle size distribution obtained from HRTEM results for (b) ThO_2 sample 1, (c) ThO_2 sample 2, (d) ThO_2 annealed at 400°C and (e) ThO_2 annealed at 800°C.

The particle size was estimated from the XRD data using the Scherrer equation and TOPAS 4.2 software. The full width at half-maximum (FWHM) of the (111) and (200) diffraction lines were used to determine the crystallite sizes with the Scherrer equation:

$$D_{hkl} = \frac{K\lambda}{\beta_{hkl}(2\theta)\cos(\theta)}, \quad (1)$$

where λ is the wavelength; K is the coefficient of anisotropy that is generally set to be 1.0 for spherical particles; θ is the scattering angle in radians; β_{hkl} is full width at half-maximum (FWHM) for diffraction peak expressed in radians. Line profiles for (111) and (200) reflections were fit to pseudo-Voigt functions. Instrumental broadening was taken into account when calculating the particle size by direct subtraction from the FWHM values. For a full-profile analysis of the diffraction patterns, and further unit cell parameter calculation, TOPAS 4.2 software was applied. Fourth-order Chebyshev polynomials with a reciprocal term were used to fit the background. The overall fitting was performed using the fundamental parameter approach. To estimate the average particle size and distribution from HRTEM images, sets of >200 particles were used.

The XRD pattern of the sample, prepared by drying initial Th(IV) precipitate at 40°C (sample 1), possesses broad diffraction peaks that indicates poor crystallinity (Figure S1, blue line). At the same time, the positions of the diffraction peaks are close to those of bulk ThO_2. The XRD pattern of the sample obtained by drying initial Th(IV) precipitate at 150°C (sample 2) clearly shows diffraction peaks

in correspondence of the ThO_2 fluorite phase (Figure S1, red line). The average NPs size estimated with the Scherrer equation is 2.0 ± 0.2 nm for sample 1 and 3.8 ± 0.4 nm for sample 2. Increasing the drying temperature from 40 °C to 150 °C induces the increase of crystallites size. Possible reasons of ThO_2 crystallites growing at 150 °C were previously discussed.[1] Annealing of the sample 1 at 400 °C, 800 °C or 1200 °C for 4 h results in a systematic increase in NPs size to 5.8 ± 0.6, 34 ± 3 and >100 nm respectively. The sample annealed at 1200 °C could be considered as a bulk ThO_2. Comparison of the sample sizes obtained from various methods and their structural characteristics are presented in the table S1. The particle size distribution obtained from the HRTEM data could be described by a unimodal lognormal function (Fig. 1b,c,d,e, inserts).

Table S1. Samples list, synthesis conditions and NPs size estimation from XRD and HRTEM.

**Sample**	**Synthesis conditions**	**Size from XRD (Scherrer equation), nm**	**Size from XRD (TOPAS software), nm**	**Size from HRTEM, nm**	**Unit cell parameter, Å**	**Microstrain value**
ThO_2 dried 40 °C sample 1	Precipitation from 0.1 M $Th(NO_3)_4$ and 3M NaOH, drying 40°C in air	2.0* ± 0.2	**-**	2.7 ± 0.4	-	-
ThO_2 dried 150 °C sample 2	Precipitation from 0.1 M $Th(NO_3)_4$ and 3M NaOH, drying 150°C in air	3.8 ± 0.4	3.8 ± 0.1	4.0 ± 0.6	5.626 ± 0.006	0.28 ± 0.06
ThO_2 annealed 400 °C	annealing of dried powder at 400°C in air	5.8 ± 0.6	6.9 ± 0.2	4.8 ± 0.9	5.613 ± 0.002	0.18 ± 0.02
ThO_2 annealed 800 °C	annealing of dried powder at 800°C in air	34 ± 3	32.0 ± 0.6	24 ± 8	5.6012 ± 0.0002	0.029 ± 0.003
ThO_2 annealed 1200 °C	annealing of dried powder at 1200°C in air	> 100	-	-	-	-

*sample showing poor crystallinity. It can be considered at the edge between X-ray amorphous and crystalline

PDF analysis and fitting

In Figure S2, Th – O and Th – Th distances from ThO_2 fluorite structure are superimposed to PDF data of bulk ThO_2 to facilitate peak assignment. The first three Th – O distances appear as well-distinguished peaks, while for *r* above 7 A they appear as small shoulders at the bottom of Th –Th peaks. Above 6.5 Å all peaks correspond to Th – Th pairs.

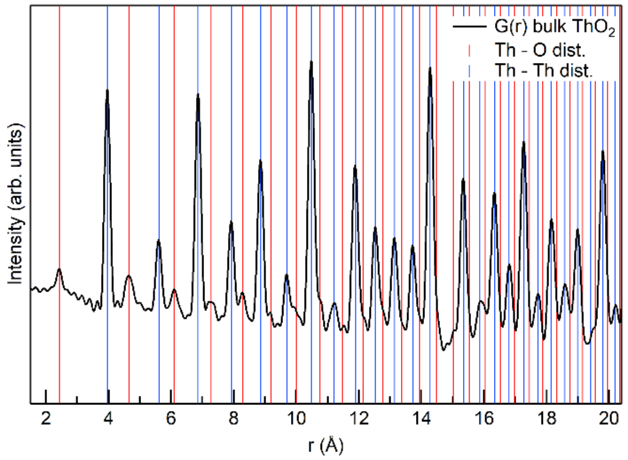

Figure S2: The experimental PDF of ThO_2 bulk is compared to Th – O (red vertical sticks) and Th – Th (blue vertical sticks) distances as extrapolated from ThO_2 structure.

Figure S3 shows the experimental shifts of the peaks between sample 1 and 2 with respect to bulk ThO_2. Both samples show a shift of peaks to higher distances. The trend for sample 2 is well approximated by a linear fit, which indicate a lattice expansion.[2] The behaviour of sample 1 is more complex: certain Th – Th peaks deviate substantially from a linear trend.

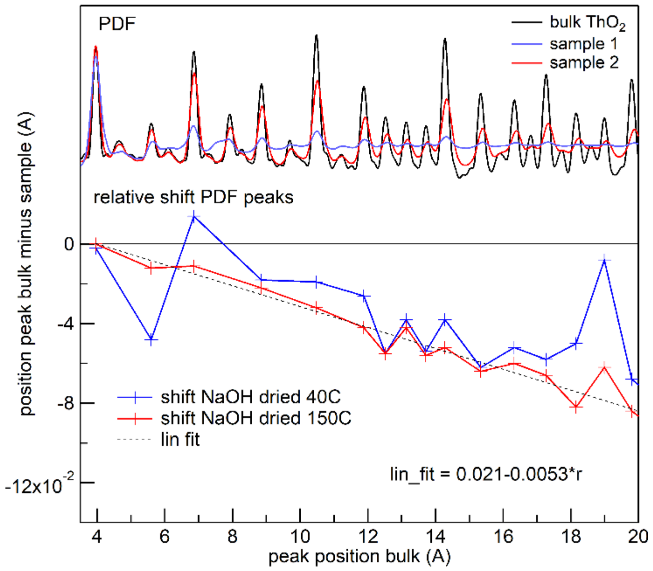

Figure S3: Top: PDF data rescale. Bottom: shift of PDF peaks for sample 1 and 2 relative to bulk ThO_2.

Figure S4 compares the lognormal distributions obtained from fitting of sample 1 (fit 1 and 2) and sample 2 with the size of real structures < 1.5 nm that can be cut out of a chunk of ThO_2. Structures differ for the number of Th atoms, i.e. we neglect the addition of only O atoms.

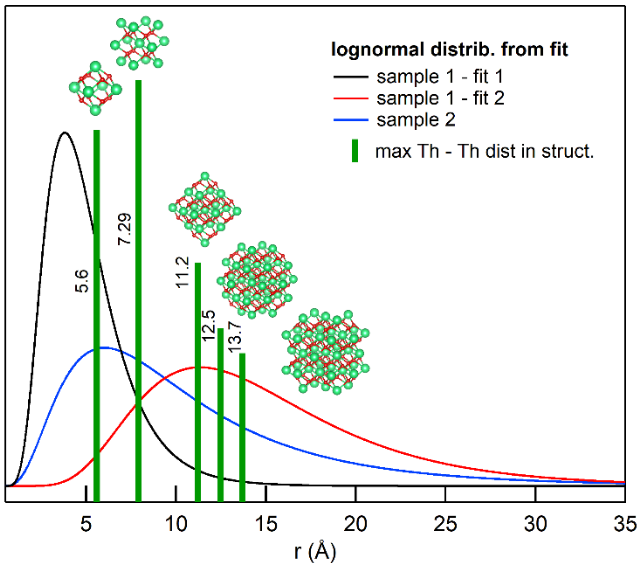

Figure S4: lognormal distributions from fits of sample 1 and sample 2 compared with real structures that can be cut out of a chunk of ThO_2. The longer Th – Th distance in the structure defines the size.

List of NP structure considered in fitting:

1. 0.56 nm
2. 1.0 nm
3. 1.12 nm - octahedral
4. 1.37 nm
5. 1.68 nm - octahedral
6. 1.75 nm
7. 2.0 nm
8. 2.1 nm
9. 2.24 nm - octahedral
10. 2.5 nm
11. 2.75 nm
12. 3.0 nm
13. 3.8 nm
14. 4.5 nm
15. 5.0 nm
16. 5.6 nm

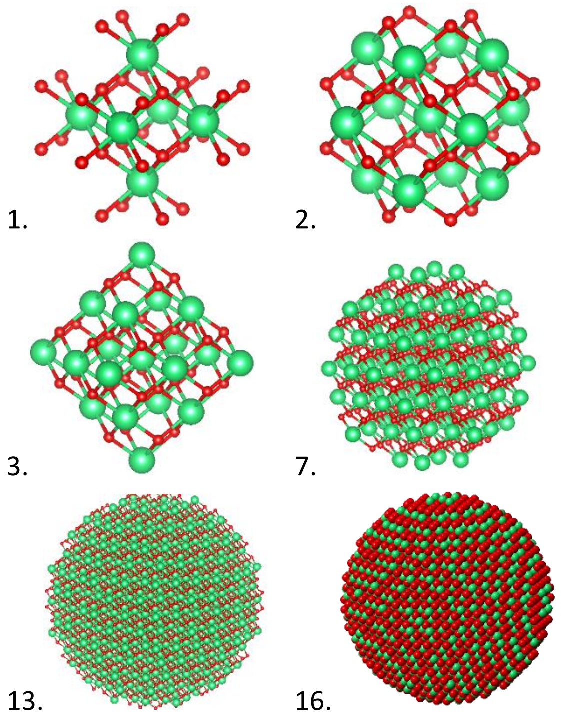

The comparison between fitting sample 1 with only NP structures and by mixing NP structures below 1.5 nm and a lognormal distribution is shown in Figure S5 and Table S2. The results are of similar quality.

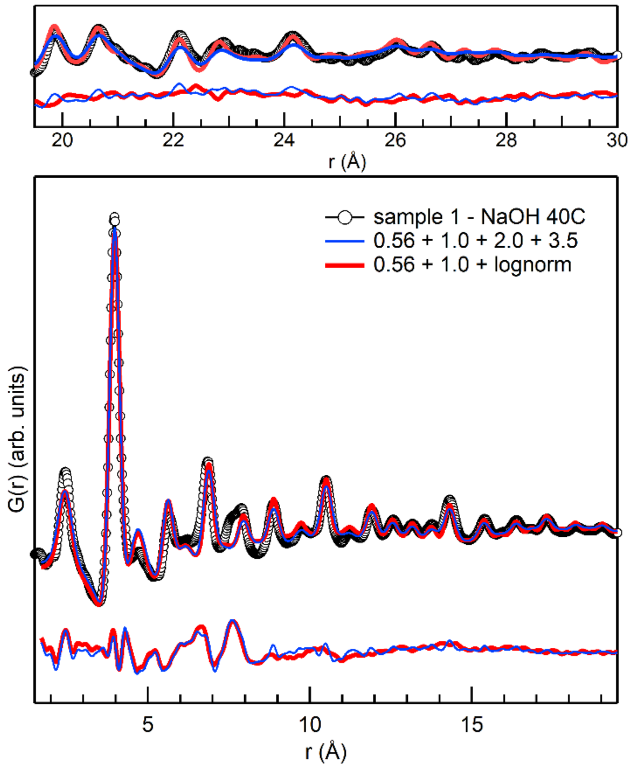

Figure S5: Experimental data of sample 1 compared with the fit with only NP structures (blues line) and the fit mixing NP structures below 1.5 nm and a lognormal distribution (red line). Residuals are shown below the data and the fits. Upper panel reports the high *r* range.

Table S2: results of fitting sample 1 with only NP structures or with a mix of NP structures and a lognormal distribution

NP	Scale factor	concentr.	*a**exp. coeff.	P_{size}	P_{sig}^2	Th U_{iso}	O U_{iso}	R_w
Sample 1 – fit with NP structures								
0.56 nm	0.60	61.3%	5.614	-	-	0.0133	0.047	0.21
1.0 nm	0.24	24.5%	5.580	-	-			
2.0 nm	0.072	7.4%	5.601	-	-			
3.5 nm	0.067	6.8%	5.619	-	-			
Sample 1 – fit NPs structures + lognormal distribution								
0.56 nm	0.66	-	5.619	-	-	0.0168	0.033	0.20
1.0 nm	0.26	-	5.601	-	-			
lognorm	0.13	-	5.616	1.0	0.6	0.0074	5.304	

FDMNES simulations

Figure S5 Reports the simulations of the Th M_4 and M_5 XANES of bulk ThO_2. The simulations are plotted with the Fermi energy as reference. The total f-DOS is shown in black thin line, the M_4 and M_5 simulated spectra are in red and blue respectively, the experimental data for bulk ThO_2 are in black symbols. The simulated spectra obeys different selection rules resulting in different structures appearing in the spectrum and different relative intensities.

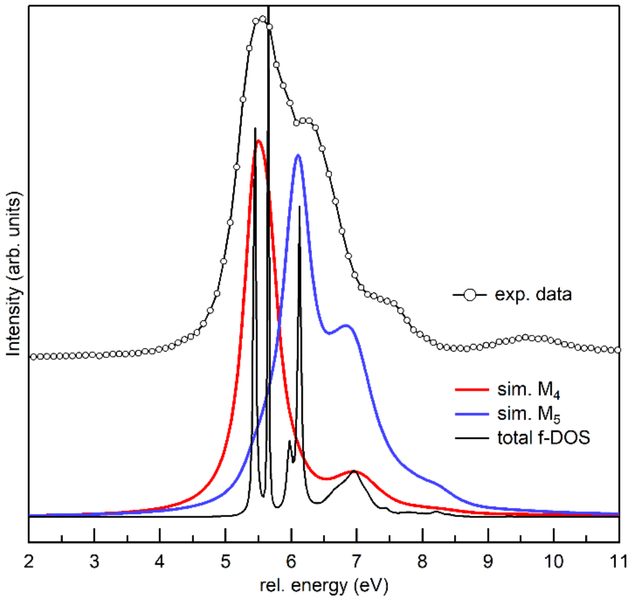

Figure S6: Comparison of FDMNES simulations of Th M_4 and M_5 XANES for bulk ThO_2. The total f-DOS is common to both simulations. The comparison shows the different selection rules operating in the two edges and shows that the second group of features in the f-DOS is selected only for M_5 edge.

An example of the input file used for FDMNES simulations is provided below:

```
Filout
R5_scf_so_scrn1_exc_coop_allDOS/out

Edge
M4

Range
-5. 0.1 0. 0.025 15. 0.5 40

Radius
5

SCF

P_self
0.01

Screening
1

Relativism
Spinorbit
Density_all
Excited

Atom
90 3 5 3 0.00 6 2 2.00 7 0 2.00
```

```
 8 2 2 0 2.00 2 1 4.00

Spgroup
 Fm-3m

COOP_atom
1

crystal
    5.6032  5.6032  5.6032  90. 90. 90.
1    0.00000    0.00000    0.00000
2    0.25000    0.25000    0.25000

Convolution
Gamma_hole
0.2
Gamma_max
0.5

End
```

References

[1] T. V. Plakhova, A. Yu. Romanchuk, D. V. Likhosherstova, A. E. Baranchikov, P. V. Dorovatovskii, R. D. Svetogorov, T. B. Shatalova, T. B. Egorova, A. L. Trigub, K. O. Kvashnina, V. K. Ivanov, S. N. Kalmykov, *J. Phys. Chem. C* **2019**, *123*, 23167–23176.

[2] B. Gilbert, *Science* **2004**, *305*, 651–654.
